# Supplementary material for: Life satisfaction and parental support among secondary school students in Urumqi: the mediation of physical activity
Source: PeerJ. 2022 Nov 10;10:e14122. doi: 10.7717/peerj.14122 (PMC9657177; doi:10.7717/peerj.14122)
Supplement: Supplemental Information 2 — A national and international public scale, the details of which can be found in the questionnaire (PAQ-CN). [file peerj-10-14122-s002.docx]

**Activity Questionnaire for Children and Adolescents (PAQ-CN)**

**A1** What activities did you do in the last week? How many times have you done each activity?

(Please type "✔" under the appropriate option)

| Activities | ①0 times/week | ②1-2 times/week | ③3-4 times/week | ④5-6 times/week | ⑤7 times and more/week |
| --- | --- | --- | --- | --- | --- |
| jump rope |  |  |  |  |  |
| Kick the shuttlecock |  |  |  |  |  |
| roller skating |  |  |  |  |  |
| Chase games |  |  |  |  |  |
| Take a walk or walk |  |  |  |  |  |
| Cycling |  |  |  |  |  |
| jogging |  |  |  |  |  |
| Jumping aerobics |  |  |  |  |  |
| swim |  |  |  |  |  |
| Play baseball/softball |  |  |  |  |  |
| dance |  |  |  |  |  |
| Play table tennis |  |  |  |  |  |
| Play badminton |  |  |  |  |  |
| Play skateboarding |  |  |  |  |  |
| Play soccer |  |  |  |  |  |
| Play basketball |  |  |  |  |  |
| Play tennis |  |  |  |  |  |
| Play volleyball |  |  |  |  |  |
| Martial arts training |  |  |  |  |  |
| Ice skating |  |  |  |  |  |
| skiing |  |  |  |  |  |
| Ice hockey |  |  |  |  |  |

**A2-A8** In the last 7 days, select the scenario that best matches the following options:

| A2 How have you spent the last week in physical education (e.g. running, playing, etc.)? | ①I don't like physical education;②I don't exercise much;③Sometimes it's like this;④It's often the case;⑤It's always been so. |
| --- | --- |
| A3 How do you spend your recess during the school week? | ①Sit and chat or study;②Walk around;③Outdoor low-intensity activities;④Outdoor medium-intensity activities;⑤Have fun outside |
| A4 What will you do during your lunch break (except for lunch) for the past week? | ①Sit and chat or study;②Walk around;③Outdoor low-intensity activities;④Outdoor medium-intensity activities;⑤Have fun outside |
| A5 In the past week, have you participated in activitiesafter school except weekends(basketball, etc.)? | ①No;②1time/week;③2-3 times/week;④4 times/ week;⑤5 times and o/week |
| A6 Have you been exercising at night for the past week(such as basketball, dancing, etc.)? | ①No;②1time/week;③2-3times/week;④4-5 times/week;⑤6-7 times and above/week |
| A7 How many activities did you do last weekend? | ①No;②1time/week;③2-3times/week;④4-5 times/week;⑤6 times or more/week |
| A8Which of the following matches your sport last week? | ①My time is almost occupied and I rarely exercise;②I occasionally (1-2 times) do more than 30 minutes of exercise (such as cycling, running, etc.);③I sometimes (3-4 times) do more than 30 minutes of exercise;④I do exercise for more than 30 minutes frequently (5-6 times);⑤I do more than 30 minutes of exercisealmost every day (7 times or more) (such as cycling, running, etc.) |

**A9** How often did you perform activities (such as basketball, soccer, badminton, etc.) for 30 minutes or more a day last week?

|  | ①No | ②Occasionally | ③General | ④Often | ⑤Very frequent |
| --- | --- | --- | --- | --- | --- |
| Monday |  |  |  |  |  |
| Tuesday |  |  |  |  |  |
| Wednesday |  |  |  |  |  |
| Thursday |  |  |  |  |  |
| Friday |  |  |  |  |  |
| Saturday |  |  |  |  |  |
| Sunday |  |  |  |  |  |
