# Supplementary material for: Life satisfaction and parental support among secondary school students in Urumqi: the mediation of physical activity
Source: PeerJ. 2022 Nov 10;10:e14122. doi: 10.7717/peerj.14122 (PMC9657177; doi:10.7717/peerj.14122)
Supplement: Supplemental Information 3 — A national and international public scale, the details of which can be found in the questionnaire (ACTS-CN). [file peerj-10-14122-s003.docx]

**Child Adolescent Parent Support Scale (ACTS-CN)**

**1.**Which of the following descriptions of your mother do you think is more reasonable?

(The table is only for the mother's situation)

|  | Strongly disagree | Disagree | Identification | Strongly agree |
| --- | --- | --- | --- | --- |
| 1.Mother usually exercises on her own. |  |  |  |  |
| 2.My mother usually exercises with me (such as walking, biking, etc.) |  |  |  |  |
| 3.When my mother exercises, she will ask me to exercise with her. |  |  |  |  |
| 4.My mother will send me to places where I can do activities (such as parks, hobby classes, etc.). |  |  |  |  |
| 5.My mother will enroll me in  interest classes, clubs, etc. (such as basketball, dancing, etc.). |  |  |  |  |
| 6.My mother keeps an eye on me when I am exercising. |  |  |  |  |
| 7.My mother allows me to watch TV without limiting the length of time. |  |  |  |  |
| 8.My mother still allows me to use the computer outside of my studies, and there is no limit to the number of hours I can use it. |  |  |  |  |
| 9.My mother allows me to play video games / physical games, no limit on the length of time. |  |  |  |  |

**2.**Which of the following descriptions of your father do you think is more reasonable?

(The table is only for the father's situation)

|  | Strongly disagree | Disagree | Identification | Strongly agree |
| --- | --- | --- | --- | --- |
| 1.Father usually exercises on his own. |  |  |  |  |
| 2.My father usually exercises with me (such as walking, biking, etc.) |  |  |  |  |
| 3.When my father exercises, he will ask me to exercise with him. |  |  |  |  |
| 4.My father will send me to places where I can do activities (such as parks, hobby classes, etc.). |  |  |  |  |
| 5.My father will enroll me in interest classes, clubs, etc. (such as basketball, dancing, etc.). |  |  |  |  |
| 6.My father keeps an eye on me when I am exercising. |  |  |  |  |
| 7.My father allows me to watch TV without limiting the length of time. |  |  |  |  |
| 8.My father still allows me to use the computer outside of my studies, and there is no limit to the number of hours I can use it. |  |  |  |  |
| 9.My father allows me to play video games / physical games, no limit on the length of time. |  |  |  |  |
