# Supplementary material for: Life satisfaction and parental support among secondary school students in Urumqi: the mediation of physical activity
Source: PeerJ. 2022 Nov 10;10:e14122. doi: 10.7717/peerj.14122 (PMC9657177; doi:10.7717/peerj.14122)
Supplement: Supplemental Information 4 — A national and international public scale, the details of which can be found in the questionnaire (ASLSS). [file peerj-10-14122-s004.docx]

Adolescent Student Life Satisfaction Scale (ASLSS)

| Projects | Fully compliant | Confor-mity | Kind of in line | Maybe | Kind of not in line | Not compliant | Not at all |
| --- | --- | --- | --- | --- | --- | --- | --- |
| 1. My friends all respect me. |  |  |  |  |  |  |  |
| 1. I have a lot of friends. |  |  |  |  |  |  |  |
| 1. My friends will help me if I need it. |  |  |  |  |  |  |  |
| 1. My friends treat me well. |  |  |  |  |  |  |  |
| 1. I have a lot of authority among my own peers. |  |  |  |  |  |  |  |
| 1. I want to make friends who are different from what I am now. |  |  |  |  |  |  |  |
| 1. I have a lot of fun things to do with my friends. |  |  |  |  |  |  |  |
| 1. I love being with my parents. |  |  |  |  |  |  |  |
| 1. My family is a happy family. |  |  |  |  |  |  |  |
| 1. Most of the time I like the way parents are educated. |  |  |  |  |  |  |  |
| 1. My family get along very well together. |  |  |  |  |  |  |  |
| 1. My parents can treat me equally. |  |  |  |  |  |  |  |
| 1. My family members   are friendly to each other. |  |  |  |  |  |  |  |
| 1. I was able to have a pleasant conversation with my parents. |  |  |  |  |  |  |  |
| 1. I love school. |  |  |  |  |  |  |  |
| 1. I like the school life. |  |  |  |  |  |  |  |
| 1. I like the school's activities. |  |  |  |  |  |  |  |
| 1. My life at school was fun. |  |  |  |  |  |  |  |
| 1. I don't feel well at school. |  |  |  |  |  |  |  |
| 1. There are a lot of things I don't like at school. |  |  |  |  |  |  |  |
| 1. I want to live somewhere else, not where I am now. |  |  |  |  |  |  |  |
| 1. There are many unsatisfactory things around me that I live around. |  |  |  |  |  |  |  |
| 1. Where I live, social security is good. |  |  |  |  |  |  |  |
| 1. The place where I live, the social atmosp-   here is good. |  |  |  |  |  |  |  |
| 1. The world in which we live is peaceful and tranquil. |  |  |  |  |  |  |  |
| 1. Basically no one forced me to do things I didn't like to do. |  |  |  |  |  |  |  |
| 1. Basically,I can do what I want. |  |  |  |  |  |  |  |
| 1. Basically,I have the freedom to make my own choices. |  |  |  |  |  |  |  |
| 1. I can do what I like to do in my spare time. |  |  |  |  |  |  |  |
| 1. Basically,no one int-   erferes with my life. |  |  |  |  |  |  |  |
| 1. I achieve desired academic success. |  |  |  |  |  |  |  |
| 1. I am satisfied with my academic status. |  |  |  |  |  |  |  |
| 1. Compared to my classmates,I am more fully developed at school. |  |  |  |  |  |  |  |
| 1. I get more honors in school compared to my classmates. |  |  |  |  |  |  |  |
| 1. I feel dignified among my peers. |  |  |  |  |  |  |  |
| 1. I have a great sense of accomplishment academically. |  |  |  |  |  |  |  |
