# Supplementary material for: Life satisfaction and parental support among secondary school students in Urumqi: the mediation of physical activity
Source: PeerJ. 2022 Nov 10;10:e14122. doi: 10.7717/peerj.14122 (PMC9657177; doi:10.7717/peerj.14122)
Supplement: Supplemental Information 5 — The Chinese version of the questionnaire can be found in this scale. [file peerj-10-14122-s005.docx]

**儿童青少年身体活动调查问卷（PAQ-CN）**

**A1 上一周，您做过哪些活动？每项活动做过几次？ （请在相应选项下打“✔ ”）**

| **活动项目** | **①0 次/周** | **②1-2 次/周** | **③3-4 次/周** | **④5-6 次/周** | **⑤7 次及以上/周** |
| --- | --- | --- | --- | --- | --- |
| 跳绳 |  |  |  |  |  |
| 踢毽子 |  |  |  |  |  |
| 轮滑 |  |  |  |  |  |
| 追逐类游戏 |  |  |  |  |  |
| 散步或健步走 |  |  |  |  |  |
| 骑自行车 |  |  |  |  |  |
| 慢跑 |  |  |  |  |  |
| 跳健美操 |  |  |  |  |  |
| 游泳 |  |  |  |  |  |
| 打棒球/垒球 |  |  |  |  |  |
| 跳舞 |  |  |  |  |  |
| 打乒乓球 |  |  |  |  |  |
| 打羽毛球 |  |  |  |  |  |
| 玩滑板 |  |  |  |  |  |
| 踢足球 |  |  |  |  |  |
| 打篮球 |  |  |  |  |  |
| 打网球 |  |  |  |  |  |
| 打排球 |  |  |  |  |  |
| 武术训练 |  |  |  |  |  |
| 溜冰运动 |  |  |  |  |  |
| 滑雪运动 |  |  |  |  |  |
| 冰球运动 |  |  |  |  |  |

**A2-A8 过去7天里，请选择下列选项中最符合的情况：**

| A2近一周，您是如何度过体育课的（如跑步、玩耍等）？ | ①我不喜欢体育课②几乎没怎么运动③有时候是这样  ④常常是这样⑤一直是这样 |
| --- | --- |
| A3近一周，您在校期间如何度过课间休息？ | ①坐着聊天或学习②四处走动③在室外低强度活动  ④在室外中强度活动⑤在外面尽情玩 |
| A4近一周，您在午休（吃午饭除外）会做什么？ | ①坐着聊天或学习②四处走动③在室外低强度活动  ④在室外中强度活动⑤在外面尽情玩 |
| A5近一周，您放学后（除周末）参加活动（篮球等）吗？ | ①没有②1次/周③2-3次/周  ④4次/周⑤5次及上/周 |
| A6近一周，您晚上进行活动锻炼（如篮球、跳舞等）吗？ | ①没有②1次/周③2-3次/周  ④4-5次/周⑤6-7次及以上/周 |
| A7上个周末，您进行过多少次活动锻炼？ | ①没有②1次/周③2-3次/周  ④4-5次/周⑤6次及以上/周 |
| A8以下哪项符合您上周运动的情况？ | ①我时间几乎被占用，很少运动；②我偶尔(1-2次)做30分钟以上运动(如骑车、跑步等) ；③我有时(3-4次)做30分钟以上的运动；  ④我频繁地(5-6次)做30分钟以上的运动；⑤我几乎每天(7次及以上) 做30分钟以上的运动(如骑车、跑步等) |

**A9** **上一周，您每天进行30分钟及以上的活动（如篮球、足球、羽毛球等）的频率？**

|  | ①没有 | ②偶尔 | ③一般 | ④经常 | ⑤很频繁 |
| --- | --- | --- | --- | --- | --- |
| 周一 |  |  |  |  |  |
| 周二 |  |  |  |  |  |
| 周三 |  |  |  |  |  |
| 周四 |  |  |  |  |  |
| 周五 |  |  |  |  |  |
| 周六 |  |  |  |  |  |
| 周日 |  |  |  |  |  |
