# Supplementary material for: Life satisfaction and parental support among secondary school students in Urumqi: the mediation of physical activity
Source: PeerJ. 2022 Nov 10;10:e14122. doi: 10.7717/peerj.14122 (PMC9657177; doi:10.7717/peerj.14122)
Supplement: Supplemental Information 6 — The Chinese version of the questionnaire can be found in this scale. [file peerj-10-14122-s006.docx]

**儿童青少年家长支持量表（ACTS-CN）**

**1.您认为下列对母亲的描述，哪项比较合理？（该表格仅针对母亲情况回答）**

|  | ①非常不认同 | ②不认同 | ③认同 | ④非常认同 |
| --- | --- | --- | --- | --- |
| 1.母亲平时会自己进行运动锻炼 |  |  |  |  |
| 2.母亲平时会与我一起运动（如散步、骑车等） |  |  |  |  |
| 3.母亲运动时，会叫我和她一起运动 |  |  |  |  |
| 4.母亲会送我到活动的场所（如公园、兴趣班等） |  |  |  |  |
| 5.母亲会为我报名兴趣班、俱乐部等（如篮球、跳舞等） |  |  |  |  |
| 6.我在运动锻炼时，母亲会在旁边关注着我 |  |  |  |  |
| 7.母亲允许我看电视，不限制时长 |  |  |  |  |
| 8.学习之外，母亲仍允许我使用电脑，且不限制时长 |  |  |  |  |
| 9.母亲允许我玩视频类/体感类游戏，不限制时长 |  |  |  |  |

**2.您认为下列对父亲的描述，哪项比较合理？（该表格仅针对父亲情况回答）**

|  | ①非常不认同 | ②不认同 | ③认同 | ④非常认同 |
| --- | --- | --- | --- | --- |
| 1.父亲平时会自己进行运动锻炼 |  |  |  |  |
| 2.父亲平时会与我一起运动（如散步、骑车等） |  |  |  |  |
| 3.父亲运动时，会叫我和他一起运动 |  |  |  |  |
| 4.父亲会送我到活动的场所（如公园、兴趣班等） |  |  |  |  |
| 5.父亲会为我报名兴趣班、俱乐部等（如篮球、跳舞等） |  |  |  |  |
| 6.我在运动锻炼时，父亲会在旁边关注着我 |  |  |  |  |
| 7.父亲允许我看电视，不限制时长 |  |  |  |  |
| 8.学习之外，父亲仍允许我使用电脑，且不限制时长 |  |  |  |  |
| 9.父亲允许我玩视频类/体感类游戏，不限制时长 |  |  |  |  |
