# Supplementary material for: Life satisfaction and parental support among secondary school students in Urumqi: the mediation of physical activity
Source: PeerJ. 2022 Nov 10;10:e14122. doi: 10.7717/peerj.14122 (PMC9657177; doi:10.7717/peerj.14122)
Supplement: Supplemental Information 7 — The Chinese version of the questionnaire can be found in this scale. [file peerj-10-14122-s007.docx]

**青少年学生生活满意度量表（ASLSS）**

| 项目 | 完全符合 | 符合 | 有点符合 | 说不定 | 有点不符合 | 不符合 | 完全不符合 |
| --- | --- | --- | --- | --- | --- | --- | --- |
| 1.我的朋友都很尊重我 |  |  |  |  |  |  |  |
| 2.我有很多朋友 |  |  |  |  |  |  |  |
| 3.如果我需要，朋友们都会帮助我 |  |  |  |  |  |  |  |
| 4.我的朋友们对我很好 |  |  |  |  |  |  |  |
| 5.我在自己的同伴中很有威信 |  |  |  |  |  |  |  |
| 6.我希望结交与现在不同的朋友 |  |  |  |  |  |  |  |
| 7.我与我的朋友在一起有很多趣事 |  |  |  |  |  |  |  |
| 8.我喜欢和我的父母在一起 |  |  |  |  |  |  |  |
| 9.我的家庭是一个幸福的家庭 |  |  |  |  |  |  |  |
| 10.大多数时候我喜欢家长的教育方式 |  |  |  |  |  |  |  |
| 11.我的家人在一起相处很和睦 |  |  |  |  |  |  |  |
| 12.我的父母能平等地对待我 |  |  |  |  |  |  |  |
| 13.我的家庭成员之间相互讲话很友善 |  |  |  |  |  |  |  |
| 14.我和我的父母在一起能愉快地交谈 |  |  |  |  |  |  |  |
| 15.我喜欢学校 |  |  |  |  |  |  |  |
| 16.我喜欢学校的生活 |  |  |  |  |  |  |  |
| 17.我喜欢学校的活动 |  |  |  |  |  |  |  |
| 18.我在学校的生活很有趣 |  |  |  |  |  |  |  |
| 19.我在学校里感到不舒服 |  |  |  |  |  |  |  |
| 20.学校的很多事情我都不喜欢 |  |  |  |  |  |  |  |
| 21.我想住在别的地方，而不是现在的地方 |  |  |  |  |  |  |  |
| 22.我生活的周围有许多不如意的事情 |  |  |  |  |  |  |  |
| 23.我生活的地方，社会治安好 |  |  |  |  |  |  |  |
| 24.我生活的地方，社会风气好 |  |  |  |  |  |  |  |
| 25.我们生存的世界是和平安宁的 |  |  |  |  |  |  |  |
| 26基本上没人强迫我做不喜欢做的事情 |  |  |  |  |  |  |  |
| 27.基本上我都能按照自己的愿望行事 |  |  |  |  |  |  |  |
| 28.基本上我有自主选择的自由 |  |  |  |  |  |  |  |
| 29.我在课余时间能做自己喜欢做的事 |  |  |  |  |  |  |  |
| 30.基本上没有人干涉我的生活 |  |  |  |  |  |  |  |
| 31.我在学业上取得理想的成就 |  |  |  |  |  |  |  |
| 32.我对我的学业状况满意 |  |  |  |  |  |  |  |
| 33.与同学相比，我在学校发展较全面 |  |  |  |  |  |  |  |
| 34.与同学相比，我在学校得到荣誉较多 |  |  |  |  |  |  |  |
| 35.我觉得自己在同伴中很有面子 |  |  |  |  |  |  |  |
| 36.我在学业上很有成就感 |  |  |  |  |  |  |  |
